# Negative screening of Fabry disease in patients with conduction disorders requiring a pacemaker

**DOI:** 10.1186/s13023-019-1140-3

**Published:** 2019-07-08

**Authors:** Ángela López-Sainz, Vicente Climent, Tomas Ripoll-Vera, Maria Angeles Espinosa, Roberto Barriales-Villa, Marina Navarro, Javier Limeres, Diana Domingo, David C. Kasper, Pablo Garcia-Pavia

**Affiliations:** 1 Department of Cardiology, Hospital Universitario Puerta de Hierro Majadahonda, Manuel de Falla, 2. Majadahonda, 28222 Madrid, Spain; 2 CIBER in Cardiovascular Diseases (CIBERCV), Madrid, Spain; 3 Cardiology Department, Hospital General Universitario de Alicante, ISABIAL – FISABIO, Alicante, Spain; 4 Cardiology Department, Hospital Universitario Son Llatzer, IdISBa, Mallorca, Spain; 5 Heart Failure and Inherited Cardiac Diseases Unit, Department of Cardiology, Hospital General Universitario Gregorio Marañón, Madrid, Spain; 6 Inherited Cardiovascular Diseases Unit, Cardiology Service, Complexo Hospitalario Universitario de A Coruña, Instituto de Investigación Biomédica de A Coruña (INIBIC) ServizoGalego de Saúde (SERGAS), Universidade da Coruña, A Coruña, Spain; 7 Department of Cardiology, Hospital Universitario Virgen de Arrixaca, El Palmar, Spain; 8 Inherited Cardiovascular Diseases Unit, Department of Cardiology, Valld’Hebron University Hospital, Barcelona, Spain; 9 Department of Cardiology, Hospital UniversitarioLa Fe, Valencia, Spain; 10 ARCHIMED Life Science, Leberstrasse 20, 1110 Vienna, Austria; 11 University Francisco de Vitoria (UFV), Pozuelo de Alarcon, Madrid, Spain

**Keywords:** Fabry disease, Conduction disease, Pacemaker, Left ventricular hypertrophy, Screening

## Abstract

Identification of Fabry disease (FD) in cardiac patients has been restricted so far to patients with left ventricular hypertrophy. Conduction problems are frequent in FD and could precede other manifestations, offering a possible earlier diagnosis.

We studied the prevalence of FD in 188 patients < 70 years with conduction problems requiring pacemaker implantation. Although classical manifestations of FD were not rare, no patient with FD was identified. Screening efforts should not be conducted in this population.

Anderson-Fabry disease (AFD) is a rare condition associated with an important diagnostic delay [1]. Contributing factors are the rarity of this disease and the lack of awareness among physicians. Early diagnosis is important in AFD because appropriate therapies seem to be more effective when initiated promptly.

AFD is an X-linked disordersecondary to mutations in the *GLA* gene, resulting in a deficiency in α-galactosidase A (α-Gal A), which leads to the abnormal accumulation of globotriaosylceramide in affected organs. At the cardiac level, patients with AFD can present with hypertrophic cardiomyopathy, microvascular angina and conduction problems/arrhythmias [2].

Efforts in the identification of AFD in cardiac patients have been restricted to screening of large series of patients with unexplained left ventricular hypertrophy, where AFD has been reported in 1–2% of individuals [3].

Although AFD patients mostly die because of cardiac problems, unexplained left ventricular hypertrophy is present in only one-half of all AFD patients. Moreover, conduction problems and arrhythmias are a frequent problem in patients with AFD and could precede other cardiac manifestations, offering a possible earlier diagnosis. Indeed, the rate of pacemaker insertion (PMI) in AFD has been described to be 25 times higher than in the general population, and the requirement of PMI has been reported to be as high as 8% in some AFD series [4, 5].

With these data, we hypothesized that AFD could be an underdiagnosed entity among patients with unexplained conduction disturbances requiring PMI, and that this group of patients could be an appropriate group to test screening strategies.

We therefore designed a multicenter, cross-sectional study to investigate the prevalence of AFD in patients with unexplained atrioventricular blockade or sinus node disease requiring PMI at < 70 years of age.

A total of 188 patients (66% males, mean age 63 ± 9 years) were identified from the existing PMI databases at 8 Spanish hospitals. All patients fulfilling inclusion criteria who underwent PMI between January 2015 and December 2016 were invited to participate. Males were screened by measuring α-Gal A activity and those with reduced levels were genetically tested for *GLA* mutations. All females were tested for mutations in *GLA*.

Baseline clinical characteristics of the cohort, stratified by sex, are summarized in Table 1.

**Table 1:** Baseline characteristics of patients with unexplained conduction disorders and who underwent pacemaker implantation screened for AFD stratified by sex

	Total (*n* = 188)	Males (*n* = 124)	Females (*n* = 64)
Age at pacemaker implantation, years	63 ± 9	64 ± 7	62 ± 8
Medical history of neuropathy, n (%)	5 (3)	4 (3)	1(2)
Medical history of renal impairment, n (%)	24 (13)	20 (16)	4 (6)
Medical history of stroke, n (%)	24 (13)	19 (15)	5 (8)
Family history of SCD, n (%)	14 (7)	8 (7)	6 (9)
Family history of HCM, n (%)	2 (1)	1 (1)	1 (2)
Family history PMI, n (%)	27 (14)	18 (15)	9 (14)
Syncope, n (%)	73 (39)	45 (36)	28 (44)
Serum creatinine (mg/dl)	1.03 ± 0.46	1.08 ± 0.44	0.94 ± 0.49
PR interval, ms	189 ± 0.44	191 ± 57	186 ± 443
QRS interval, ms	116 ± 28	120 ± 27	109 ± 286
LVH in ECG, n (%)	40 (21)	32 (26)	8 (13)
Interventricular septum, mm	12 ± 3	12 ± 3	11 ± 3
LVEF, n (%)	60 ± 10	59 ± 11	63 ± 8
α-Gal activity, μmol/L/h	3.8 ± 1.7	3.9 ± 1.7	3.6 ± 1.6

*α-Gal A* α-galactosidase A, *HCM* hypertrophic cardiomyopathy, *LVH* left ventricular hypertrophy, *LVEF* left ventricular ejection fraction, *PMI* pacemaker insertion, *SCD* sudden cardiac death

Almost one-third of our cohort presented evidence of echocardiographic alterations. Left ventricular hypertrophy on echocardiogram (≥12 mm) was found in 46% of patients. ECG signs compatible with AFD, including prolonged PR interval or left ventricle hypertrophy, were not infrequently reported (30 and 22%, respectively). Regarding other non-cardiac classical findings related to AFD, 17% of the patients showed some degree of renal impairment (defined as a glomerular filtration < 50 mL/min/1.73m^2^), and 3% reported neuropathic disease.

From the 188 patients with conduction problems undergoing PMI screened for AFD, we failed to find any patient with the condition. The mean α-Gal A enzyme activity in males was 3.8 ± 1.7 μmol/L/h, more than threefold higher than the normal cut-off value (1.2 μmol/L/h). A total of 75 individuals (11 males and 64 females) were genetically tested for mutations in the *GLA* gene. Genetic variants associated with low α-Gal A enzyme activity that are considered to be polymorphisms (e.g.,p.D313Y) were present in 1 female but no patient had a pathogenic mutation.

Reasons for the negative results of the AFD screening in this population could include relative younger age and absence of prolonged QRS interval in some of the patients studied. It has been reported that among AFD patients, those older and with longer QRS interval (> 110 ms) are at higher risk of requiring antibradycardia pacing [4]. In our cohort, the mean age was 63 years and 48% of patients exhibited a QRS interval < 110 ms.

In conclusion, although our data suggest that classical cardiac manifestations attributed to AFD are not rare in the population requiring PMI, AFD is not a frequent cause of unexplained conduction problems requiring PMI. Therefore, it seems that screening efforts should not be conducted in this patient population.

The appropriate group of patients to whom AFD screening should be performed in the cardiac setting beyond those with unexplained LV hypertrophy ≥15 mm will require further evaluation. Other possible groups of patients with cardiac disease in whom AFD screening would need to be evaluated include patients with mild LVH, those with ECG abnormalities (short PR interval, high voltages and/or inverted T waves) without overt LVH, and those with vasospastic angina.

## Data Availability

Please contact corresponding author for data requests.

## Abbreviations

AFD: Anderson-Fabry Disease
LV: Left ventricular
PMI: Pacemaker insertion
